# Flexion-extension-supination test compared to arthroscopic findings of biceps long head pathology: A physical examination that reflect anatomical evolution of human shoulder girdle

**DOI:** 10.1097/MD.0000000000029755

**Published:** 2022-07-15

**Authors:** Byung-Kook Kim, Ho-Jae Lee, Suk-Han Jung, Jinmyoung Dan

**Affiliations:** Department of Orthopaedic Surgery, Gumi CHA Medical Center, School of Medicine, CHA University, Republic of Korea

**Keywords:** arthroscopy, long head of biceps tendon, physical examination, shoulder

## Abstract

The accuracy of physical examination for diagnosing lesions of the long head of the biceps tendon (LHBT) remains unsatisfactory. The purpose of this study was to describe a new diagnostic test, the Flexion-Extension-Supination (FES) test for diagnosing lesions of the long head of biceps tendon.

A prospective study of 162 patients was performed to evaluate the diagnostic value of FES test. All the participants were evaluated on the basis of their clinical presentation, physical examination (FES test), radiologic findings and arthroscopic examination. Shoulder arthroscopy findings were used as the gold standard. To reduce the omission of the hidden lesion, LHBT was checked at the intra- and the extraarticular side via arthroscopic examination.

Surgical findings related to biceps pathology were as follows: rotator cuff tears, 89.5% (145/162); subacromial impingement, 8.6% (14/162); and biceps tendinitis, 1.9% (3/162). The prevalence of biceps pathology was 77.2% (125/162) of all arthroscopic procedures. No significant differences for LHBT lesions were observed between the FES test and the arthroscopic findings (*P* = .850). The interrater reliability of the FES test was 0.747. After excluding inconclusive results between examiners, the sensitivity, specificity, positive predictive value, and negative predictive value of the FES test were 87.9%, 66.7%, 82.9%, and 63.2%, respectively. Positive and negative likelihood ratios were 2.67 and 0.18, respectively.

The maneuvers of the FES test irritate intra- and extraarticular lesion of LHBT. The FES test is a reproducible and reliable test that can be used during physical examinations to evaluate patients with LHBT lesions.

## Introduction

Disorders of the long head of the biceps tendon (LHBT) are common and often associated with other shoulder diseases.^[1,2]^ LHBT faces extraarticular and intraarticular constraints due to the constant sliding movement of the tendon within the bicipital groove during elevation and rotation of the shoulder.^[3]^ LHBT can be damaged anywhere along its course, and its pathologic changes can manifest in different ways.^[4,5]^ Numerous studies have suggested that lesions of the LHBT can be frequently found in painful or disabled shoulders.^[6,7]^ Proper diagnosis of these lesions is critical for appropriate treatment,^[8]^ as failure to address the biceps tendon has been associated with persistent pain and reduced satisfaction following shoulder surgery.^[9–11]^

In clinical practice, physicians usually perform a comprehensive history check, and use several clinical tests and imaging modalities to diagnose LHBT pathology. Practically, many physical examinations such as Speed test, Yergason test, and the direct palpation of the bicipital groove are performed as traditional provocative maneuvers. A recent study assessed several clinical tests for LHBT pathology and reported that their sensitivities ranged from 53% to 85% with a specificity of 17% to 87%.^[12]^ Several new shoulder tests have been described and validated as additional provocative tests, in certain patient populations, but their results were still unreliable for accurate diagnosis of LHBP.^[13–15]^ We assume that these test maneuvers and assessment of LHBT lesions were not sufficient to reflect various planes of shoulder motion and various locations of LHBT lesions. And these tests have a wide spectrum of sensitivity and specificity.

In this study, biceps pathologies were checked via arthroscopic examination at both the glenohumeral joint and extraarticular space. LHBT were carefully inspected especially at the extraarticular space not to miss the hidden lesions of distal portion of bicipital groove.

The purpose of this study was to determine whether the FES test could accurately diagnose LHBT lesions in patients with shoulder pathologies. The clinical utility and the value of the FES test for LHB lesions were evaluated and compared with arthroscopic findings. LHBT were carefully inspected especially at the extraarticular space so as to not miss the hidden lesions of distal portion of bicipital groove.

Our hypothesis is that the FES test reflects the complex motions of the shoulder joint, and would detect biceps lesion more accurately in diagnosis of LHBT lesion at both the glenohumeral joint and extraarticular space.

## Material and Methods

The Institutional Review Board of CHA Medical Center reviewed and approved this study (IRB No. GM1906).

A prospective observational study of 162 consecutive patients scheduled to undergo an arthroscopic procedure for chronic shoulder pain was conducted between December 2017 and December 2019 at our institution.

Inclusion criteria were: (1) persistent pain and functional disability not responsive to adequate conservative treatment for more than 3 months; (2) patients who underwent FES test; (3) patients who had undergone arthroscopic surgery with tenodesis of long tendon of biceps at bicipital groove or exploration of bicipital groove. This study was designed to evaluate the clinical effectiveness of the examination only for LHBT lesions, and patients who underwent surgery to repair complete tears of the biceps tendon and those who had superior labrum from anterior to posterior (SLAP) lesion were excluded from this study. Additionally, patients with concomitant adhesive capsulitis, glenohumeral osteoarthritis were excluded. After applying the inclusion and exclusion criteria for the study, 162 patients who underwent arthroscopic procedures were included in this study.

### Physical Examination

Each patient underwent a standardized physical examination before surgery. Patients were evaluated by 2 examiners (H.L. and B.K.) who were unaware of their preoperative clinical and imaging data. The examiners registered the evaluation results before surgery. In all cases, physical examinations and radiologic evaluations (plain radiology and MRI) were performed within 4 weeks of the procedure.

**Figure 1.**
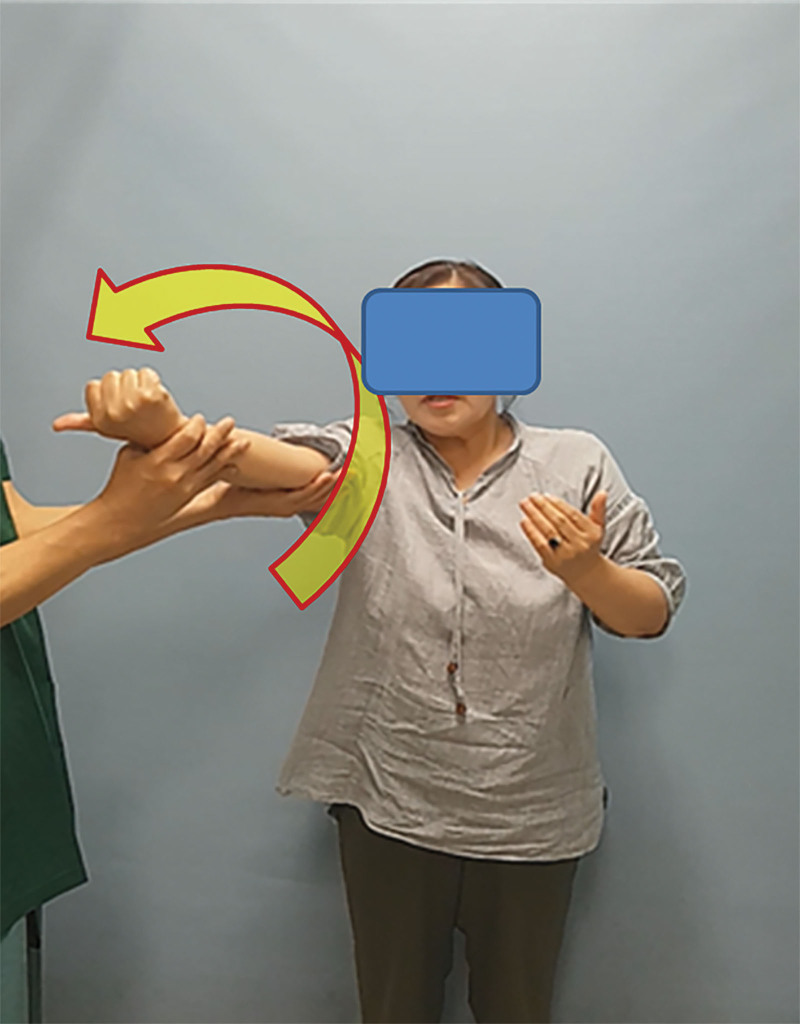
FES test is used as a provocative test to determine the presence of pain around bicipital groove caused by passive shoulder movement. To perform the FES test, the involved shoulder was flexed at 90-degrees and neutrally rotated with an extended elbow. The patient was then asked to supinate their forearms or be passively supinated by the examiner. If the patient felt pain in the bicipital groove or could not supinate fully, the test was considered as positive. The FES test result was negative if pain was not elicited in a passive forearm supination position.

Physical examination was performed while the patient was standing. To perform the FES test, the involved shoulder was flexed at 90-degrees, neutrally rotated, and extended at the elbow. The patient was then asked to supinate the forearm or be passively supinated by the examiner (Fig. 1). If the patient felt pain in the bicipital groove or could not fully supinate, the test was considered positive. The FES test results were negative if the pain was not elicited.

### Arthroscopic findings as reference

**Figure 2.**
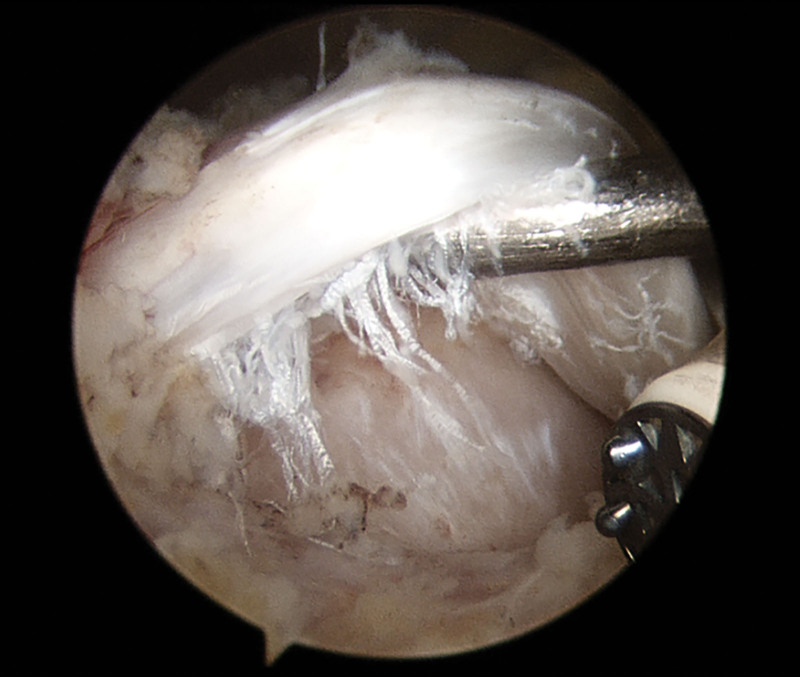
During the diagnostic phase of arthroscopy, intraarticular LHBT lesion was assessed through the posterior portal. There was a partial tear of LHBT, but not inflammatory sign around the tendon.

Arthroscopic findings were used as the gold standard to confirm pathologies of the long head of the biceps tendon. Surgery was conducted by the senior author (J.D.) who was blinded to the test results. Their biceps pathologies were checked via arthroscopic examination at both sides of the glenohumeral joint and subacromial space. intraarticular LHBT lesions were assessed through the posterior portal during the diagnostic phase of arthroscopy. The biceps tendon was probed by pulling the intraarticular portion of the tendon into the joint so that the intertubercular portion of the tendon could be visualized (Fig. 2).

**Figure 3.**
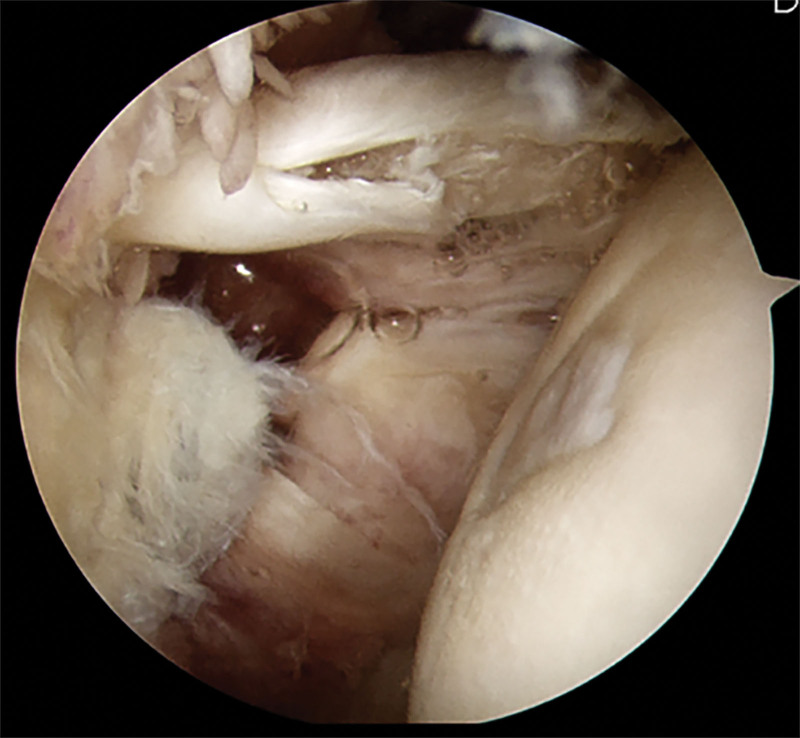
At subdeltoid space, the bicipital groove was opened with a cautery device over the whole length of the groove. After the sheath was opened, the LHBT was mobilized and visualized. Additionally, LHBT was pulled out the most distal portion of tendon to visualize other hidden lesions of distal portion of bicipital groove. There was a partial tear and fraying at whole length of LHBT. The pulled tendon from outside of bicipital groove also had degenerative changes.

After performing a standard diagnostic arthroscopy sequence, a 30-degree scope was inserted into the subacromial space and moved into the anterior compartment of the shoulder. The bicipital groove was opened with a cautery device over the middle one-third of the groove. After the sheath was opened, the LHBT was visualized and mobilized. To inspect the extraarticular LHBT over the bicipital groove, a probe was used to assess the LHBT lesion, including biceps instability. Additionally, the distal portion of the tendon was pulled out to visualize other hidden lesions of the distal portion of bicipital groove (Fig. 3). After excluding SLAP lesions or previous complete ruptures of LBHT, LHBT lesion location was categorized as intraarticular, extraarticular, or both sites. After the operation, the surgeon described every pathology and lesion site found during the operation and procedures performed per protocol.

### Statistical Analysis

Demographic data, preoperative physical examination findings, and intraoperative findings were analyzed using a standard computer program (Microsoft Excel) and SPSS (Version 20). Interrater reliabilities were calculated between examiners using Cohen kappa with 95% confidence intervals to assess the precision of each examination maneuver. For analysis, surgical findings of biceps pathology were simplified and scored in a binary fashion (positive or negative). The findings between FES tests and LHBT lesions were analyzed using the McNemar test. Prevalence, sensitivity, specificity, accuracy, and positive and negative predictive values were calculated along with their respective 95% confidence intervals.

## Results

A total of 235 patients were examined. After appropriate exclusions, 162 subjects (84 women and 78 men) aged between 40 and 80 years (mean age, 61.7, SD +/- 7.5) met the inclusion criteria for this study. These patients were admitted for rotator cuff tear (145 [89.5%]), subacromial impingement (14 [8.6%]), or biceps lesion (3 [1.9%]). Most patients could not indicate a traumatic event that caused their symptoms, although they had a chronic history of shoulder problems that exceeded 1 year.

### Primary interest: interrater reliability of the FES

In this study, 2 reviewers independently performed the FES test for every enrolled patient. There were 10 inconclusive FES test results between the 2 reviewers. The final FES test results for 152 patients were positive for 114 shoulders and negative for 38 after a conclusive decision between the 2 examiners. The interrater reliability for the test was calculated between observers using Cohen kappa with 95% confidence intervals to assess the precision of the examination maneuver. The k coefficient for the inter-observer reproducibility of the FES test was 0.747, which was considered to have substantial agreement.

During arthroscopy, we found that 125 (77.2%) patients had LHBT lesions. Partial rupture, with or without inflammation was the most common pathology. The other lesions are listed in Table 1.

**Table 1.** Description of LHBT lesion

Arthroscopic Findings	No. of patients (%)
Normal LHBT	37 (22.8%)
Abnormal LHBT	125 (77.2%)
Inflammation (tendon-only)	19
Inflammation (tendon and tendon sheath)	8
Partial tear (± inflammation)	57
Synovial remnant after previous complete rupture	2
Biceps instability (subluxation and dislocation)	39
Total number of evaluation	162

LHBT = long head of the biceps tendon.

### Secondary interest: FES test related to arthroscopic findings of LHBT lesion

We used the arthroscopic findings of the LHBT as a standard reference. There were no statistical differences for LHBT lesions between the FES test and the arthroscopic findings (*P* > 0.05). Regarding the location of the LBHT lesion, 23 cases (18%) in intraarticular side, 25 (20%) in bicipital groove, 75 (60 %) in both sides, and 2 (2%) intraarticular residual remnants after complete rupture of LHBT were found (Table 2).

**Table 2.** Locations of LHBT lesion

intraarticular lesion	23(18.4%)
Lesion in bicipital groove	27(21.6%)
Both side (include biceps instability)	75(60%)
Total number of LHBT lesions/ total evaluation	125/162

LHBT = long head of the biceps tendon.

The sensitivity and specificity of the FES test for diagnosing LHBT lesions were 82.9% and 66.7%, respectively. The positive predictive value, negative predictive value, and accuracy were 87.9%, 66.7%, and 82.9, respectively. The positive and negative likelihood ratios of the FES test were 2.67 and 0.18, respectively (Table 3).

**Table 3.** Results of comparing the FES test with arthroscopic findings for biceps lesion and diagnostic value

	Present surgically	Absent surgically	Total
Positive clinically	(a = 102)	(b = 12)	(a + b = 114)
Negative clinically	(c = 14)	(d = 24)	(c + d = 38)
Total	(a + c = 116)	(b + d = 36)	(a + b + c + d = 152)
True positive			
Positive clinical examination: Pain in bicipital groove in FES test. Patients supinated their forearms either by themselves or with the help of the examiner.			
Positive surgical findings: Inflammation (at tendon and/or tenosynovium), biceps partial tear (with or without inflammation), biceps instability (subluxed or dislocated) of the biceps tendon			
True negative			
Negative clinical examination: no symptoms.			
Negative surgical findings: grossly normal biceps tendon in arthroscopic exam.			
Prevalence	(a + c)/N	116/152	76.3%
Sensitivity	(a/a + c)	102/116	87.9%
Specificity	(d/b+ d)	24/36	66.7%
Accuracy	(a + d)/N	126/152	82.9%
Positive predictive value	(a/a + b)	102/114	89.5%
Negative predictive value	(d/c + d)	24/38	63.2%
Positive likelihood ratios	sensitivity/1-specificity		2.67
Negative likelihood ratios	1-sensitivity/specificity		0.18

FES test = Flexion-Extension-Supination test,

LHBT = long head of the biceps tendon.

There were 12 false-positive and 14 false-negative results in this study. We assumed that extraarticular impingement, such as acromial spur, might have increased the false-positive results. Interestingly, patients with intraarticular biceps residual remnant after previous biceps rupture had positive FES results; the remnant might have caused irritation in the BG with a positive FES test. In addition, some factors, such as previous medication and conservative treatment for accompanying shoulder lesions, might have led to false-negative results.

## Discussion

The FES test is a provocative test to determine the presence of pain around the bicipital groove caused by passive shoulder movement. The passive moving LHBT against the humeral head and bicipital wall can irritate biceps lesions further and cause pain on the anterior aspect of shoulder joint, especially around the bicipital groove. The main finding of this study was that the FES test can reveal the intra- and extraarticular LHBT lesions.

The comparative anatomy of the shoulder girdle has demonstrated the evolutionary positional changes of the scapula to a more dorsal plane with associated torsion of the humeral shaft.^[16,17]^ Retroversion of the proximal humerus has led to the bicipital groove no longer centered on the plane of the humeral head.^[18]^ As a consequence, the LHBT is forced to shear on the lesser tuberosity and the medial wall of the groove.^[19]^ Compared to its active function at the elbow joint, where it acts as a flexor and supinator, the function of the LHBT at the shoulder is still under debate.^[20]^ Although LHBT is considered a functionally obscure and static structure in the joint, its passive gliding movement occurs vigorously during abduction or rotation of shoulder motion during daily activity. As a consequence of various shoulder motions and their location on the proximal humerus, LHBT can be passively subluxated anteriorly and medially against the humeral head and biceps groove.^[18]^ These positional changes make the biceps highly vulnerable to damage, not only to acute trauma, but also to repetitive daily activity during aging.^[7]^

Compared to a previous study of biceps tests such as Speed or Yergason test, the FES test has higher diagnostic values. Some possible explanations for its high diagnostic value include the following: First, the initial step of the FES test was performed at 90-degree shoulder elevation with the elbow extended position. Hypertrophy, partial tear, or inflammation of the LHBT tendon prevents sliding of the tendon into the bicipital groove with elevation of the shoulder in the sagittal plane. In our study, several patients with severe inflammation or mechanical blocking of LBHT could not even extend their elbow fully because excursion of the tendon was limited. The second step of the FES test is active supination of the forearm or passive supination on the axial plane. This maneuver not only increases LHB tendon excursions, but also increases passive abutment of LHB tendon against bicipital groove and humeral head. These 2 combined passive movements of the maneuver can detect biceps insanity in 2 planes, making the test more valuable.

In addition to demonstrating the mechanism of the FES test, we simultaneously evaluated intraarticular and extraarticular LHBT findings in every case and used arthroscopic findings as a reference guide for the FES test. Therefore, we could check the hidden lesion outside the glenohumeral joint by performing an extraarticular evaluation of the LHBT tendon.

Another issue is the identification of the location of LHBT lesions that may affect the results of physical examinations. To reduce the diagnostic omissions caused by the distal extension of the lesions or the extraarticular hidden lesions, we checked LHBT at both sides of intra- and extraarticular space. In this study, more than 20% of LHBT lesion were located extraarticular site which are possible omissions in conventional arthroscopic examination. Arthroscopic assessment for detecting LHBT disorders is used as an appropriate gold standard by the majority of studies since it can recognize LHBT as a source of pain and disease.^[12,21,22]^ The technique of pulling the LHBT into the joint with an arthroscopic probe is especially well established in clinical practice. This is a standard part of diagnostic arthroscopy. However, in conventional glenohumeral arthroscopy, only 48% of the overall length of the LHBT can be visualized arthroscopically.^[4,23,24]^ The distal portion of the LHBT tendon, which is a recognized predisposing area for LHBT lesions, is not properly examined using standard arthroscopic techniques.^[10,25,26]^ Jordan and Saithna have reported that conventional glenohumeral arthroscopy allows visualization of intraarticular lesions and parts of the extraarticular lesion zone. However, it cannot adequately evaluate more distal parts of the tendon where many pathologies occur.^[27,28]^ Furthermore, in a cadaveric study, up to 48%–56% of tendons can be intraarticularly visualized. Thus, the technique of pulling the LHBT into the joint with an arthroscopic probe is inadequate for evaluating hidden lesions of the biceps.^[29]^ These findings indicate that a significant number of lesions in standard arthroscopic evaluation may be missed as a result, possibly causing a high rate of missed diagnoses.

In our study, both side lesions and extraarticular lesions accounted for 82% of the total LHBT lesions. This means that some of these lesions could be visualized more by pulling the tendon in the glenohumeral joint; however, a significant number of lesions might still be missed as a result. These missing lesions may affect the validity of the physical examinations. We assume that checking both sides of the shoulder joint can make the FES test more reliable because these procedures can reduce the possibility of omitting hidden or missing biceps lesions on the extraarticular side. The findings of this study may inform clinicians that the FES test is appropriate for discriminating LHBT pathologies and that it is helpful for the successful treatment of shoulder lesions.

This study had several strengths and limitations. The FES test can irritate pathologic LBHT on the sagittal and axial planes by 2 combined passive movements of the shoulder. It can detect the biceps problem to be more valuable. We can exclude hidden lesions caused by false positives by performing extraarticular evaluation of LHBT. However, the fact that this study was conducted in a preoperative scenario could obstruct the applicability of the results to primary care settings. Nonsurgical patients who did not require surgery were missing. This was a significant limitation in the selection of patients. Therefore, the patients included in the study might not represent the general population of patients with shoulder pain and possible LHBT disease. Additionally, we have yet to perform traditional tests such as Speed test, Yergason test, and direct palpation of bicipital groove to compare their clinical value with FES test. So, as a further study, it is required to compare diagnostic values between these traditional tests and FES test.

## Conclusions

The maneuvers of the FES test can change the excursions of LHBT, and cause passive abutment and excursions of LHBT against bicipital groove and humeral head. Thus, the FES test reflects the actual state of intraarticular and extraarticular LHBT. Clinically, the FES test is a reliable test that can be used during physical examinations to evaluate patients with LHBT lesions.

## Acknowledgements

Not applicable.

## Author contributions

Conceptualization: J Dan, BK Kim

Writing – original draft: BK Kim

Writing – review & editing: J Dan

Supervision: BK Kim

Data curation: HJ Lee, SH Jung

Formal analysis: SH Jung

Methodology: HJ Lee
